# Conservation implications for jaguars and other neotropical mammals using highway underpasses

**DOI:** 10.1371/journal.pone.0206614

**Published:** 2018-11-06

**Authors:** Alberto González-Gallina, Mircea G. Hidalgo-Mihart, Víctor Castelazo-Calva

**Affiliations:** 1 Red de Ambiente y Sustentabilidad, Instituto de Ecología A.C., El Haya, Xalapa, Veracruz, México; 2 División Académica de Ciencias Biológicas, Universidad Juárez Autónoma de Tabasco, Villahermosa, Tabasco, México; 3 Red de Biología y Conservación de Vertebrados, Instituto de Ecología A.C., El Haya, Xalapa, Veracruz, México; Smithsonian Conservation Biology Institute, UNITED STATES

## Abstract

The Nuevo Xcan-Playa del Carmen highway in Quintana Roo, bisects the vegetation corridor connecting two Jaguar Conservation Units (JCUs): Yum Balaam (north) and Sian Ka´an (south). The project´s main goal was to describe differential use of available crossing structures (wildlife underpasses and culverts) by mammals present along this highway. We set 28 camera traps along the 54km stretch of the highway covering wildlife underpasses (10), and culverts such as box culverts (9) and pipes (9) from September 2016 until March 2017. A total of 24 jaguar crossings have been recorded exclusively using wildlife underpasses, including four males and two females. At least 18 other mammal species including five of the target priority species (protected by Mexican law) were documented, all of which were native except for two invasive species. In terms of species using the crossing structures, we identified 13 species using wildlife underpasses, nine using concrete box culverts and 10 using concrete pipes. Wildlife underpasses show higher diversity values (Shannon´s exponential index = 5.8 and Inverse Simpson´s index = 4.66) compared to culverts because they allow bigger species to cross. We recommend more highways along the jaguar´s distribution should develop mitigation measures to allow for wildlife connectivity. Wildlife underpasses, along with retrofitted culverts, could help secure not only the permanence of this species by facilitating the functional connectivity between populations but have positive impacts on other neotropical mammalian fauna as well.

## Introduction

Roads and traffic affect wildlife populations by (i) increasing mortality, (ii) decreasing habitat amount and quality and (iii) fragmenting populations into smaller sub-populations each more vulnerable to local extinction than larger populations [1]. Road traffic kills hundreds of millions of animals every year [2] posing a significant threat to many species. Wildlife vehicle collisions can be one of the major causes of mortality for carnivores [3], sometimes sufficient to threaten population viability, with clear examples amongst the felids e.g. 35% mortality for Florida panther [4], 17% for the Iberian lynx [5] and 40% for ocelots in Texas [6]. Carnivore, and specifically felid, ecological traits (large body size, large home range and mobility plus low reproductive rate) and behavioral responses appear to enhance susceptibility to negative road effects [3, 7–11]. In an attempt to reduce this negative impact, wildlife crossing structures have become a common and frequently applied mitigation measure, encompassing a broad range of underpasses and overpasses. These structures help animals to cross roads safely and thus play an important role in the conservation of biodiversity by increasing the permeability of roads and reducing the risk of wildlife-vehicle collisions [12]. These structures are found widely in North America but also in the rest of the world and designed for a variety of focal taxa [11,13].

Jaguars (*Panthera onca*) have been extirpated from more than half of their original range over the last hundred years and recent conservation assessments conclude that jaguars are declining in much of their remaining range [14,15]. In Mexico, jaguars are considered an endangered species (NOM-059-SEMARNAT-2010) as they have lost most of their distribution range to humans, with only around 16% of the country’s land remaining as potentially suitable jaguar habitat [16]. Jaguar populations in Mexico are mostly concentrated in Jaguar Conservation Units (JCUs, areas with a stable prey community and known, or believed, to contain a resident population of at least 50 breeding individuals [17]) distributed throughout Mexico, still potentially connected by habitat corridors [14, 18–19]. Jaguars suffer from the negative effects of roads in a variety of ways. First, jaguars suffer an increased risk of mortality from vehicle-collisions [20], but also by hunters whose access is facilitated by roads [21]. Hunting access to new areas can also reduce prey availability [22]. Second, roads can cause significant habitat fragmentation [23]. Similar fragmentation effects have been reported on both JCUs [24] and their linking corridors in Mexico [25]. The jaguar’s status and vulnerability from negative road effects suggests an increasing necessity to facilitate connectivity between jaguar populations and reduce mortality from wildlife vehicle collisions [26]. Previous research has primarily focused on identifying the best locations for wildlife crossing structures either through habitat modelling [24, 26–27] or through road monitoring in search of road-kill hotspots as mitigation proxies [28]. Nevertheless, to our knowledge there are only two particular highway projects that built specific structures to enhance jaguar crossing, the first one in Argentina with a single overpass [29], and this project in Quintana Roo, Mexico [30].

The Nuevo Xcan-Playa del Carmen highway in Quintana Roo (hereafter NX-PC) is a 54km long highway project built in the years 2013–2014. The Environmental Impact Assessment (EIA) of the project and the Mexican Environmental Authorities identified that the new road crossed along the vegetation corridor connecting the Yum Balaam and Sian Ka´an JCUs [14] where important jaguar populations have been recorded [31–32]. Jaguar presence is not restricted exclusively to the JCUs as activity has been recorded along this corridor as well [33]. In order to maintain the connectivity of these jaguar populations, the Mexican environmental authorities mandated the construction of 28 wildlife underpasses to facilitate the safe crossing of jaguars, ocelots (*Leopardus pardalis*), margays (*Leopardus wiedii*), pumas (*Puma concolor*) and tayras (*Eira barbara*; S.G.P.A./D.G.I.R.A./D.G./9495), and potentially for the other species present in the area [34]. The road opened in November 2014, becoming the only highway project in southern Mexico where mammalian crossing structures were operating.

Monitoring needs to be an integral part of a highway mitigation project long after the measures have been in place as it allows agencies to better evaluate the performance of their mitigation investments and inform decision making with regard to planning and design of mitigation on future projects [35]. The monitoring of priority species using crossing structures, particularly jaguars along the NX-PC highway will give us feedback for further recommendations for future highway projects not only in Mexico but across Latin America. As such, our first objective in this study is to determine the use of available crossing structures by medium to large mammals with an emphasis in jaguar usage.

The use of particular wildlife crossing structures depends on the ecological adaptations of each species [36]. Different types of crossing structures are needed to increase habitat connectivity for the wide diversity of carnivore species [3]. The differential use of wildlife crossing structures (including culverts) by medium and large mammals has been observed, where larger structures generally have higher rate of use by carnivores than small ones [37]. Small-sized underpasses are usually preferred by carnivores such as bobcats (*Lynx rufus)* [38–39] or pumas [40], species that require cover or concealment and might be more tolerant to restricted spaces. It appears that carnivores that use open habitat prefer overpasses (e.g. wolves, grizzly bears) [41].

The NX-PC counts with 28 wildlife underpasses and 88 culverts, 32 box culverts and 56 pipes that could potentially be useful as crossing structures for medium and large sized mammals. Our main objective is to determine if there is a differential use of the wildlife crossing structures compared to culverts by the mammal community, with focus on jaguars. Identifying which species use them for crossing and the utility of these as wildlife underpasses is basic information that the Mexican environmental authorities should take in account for the resolution of future road projects. In México, EIAs usually recommended the use of culverts as surrogates of wildlife underpasses [42]. We want to make clear that only under certain circumstances can culverts be considered to function as wildlife crossing structures.

## Methods

### Study area

**Fig 1:**
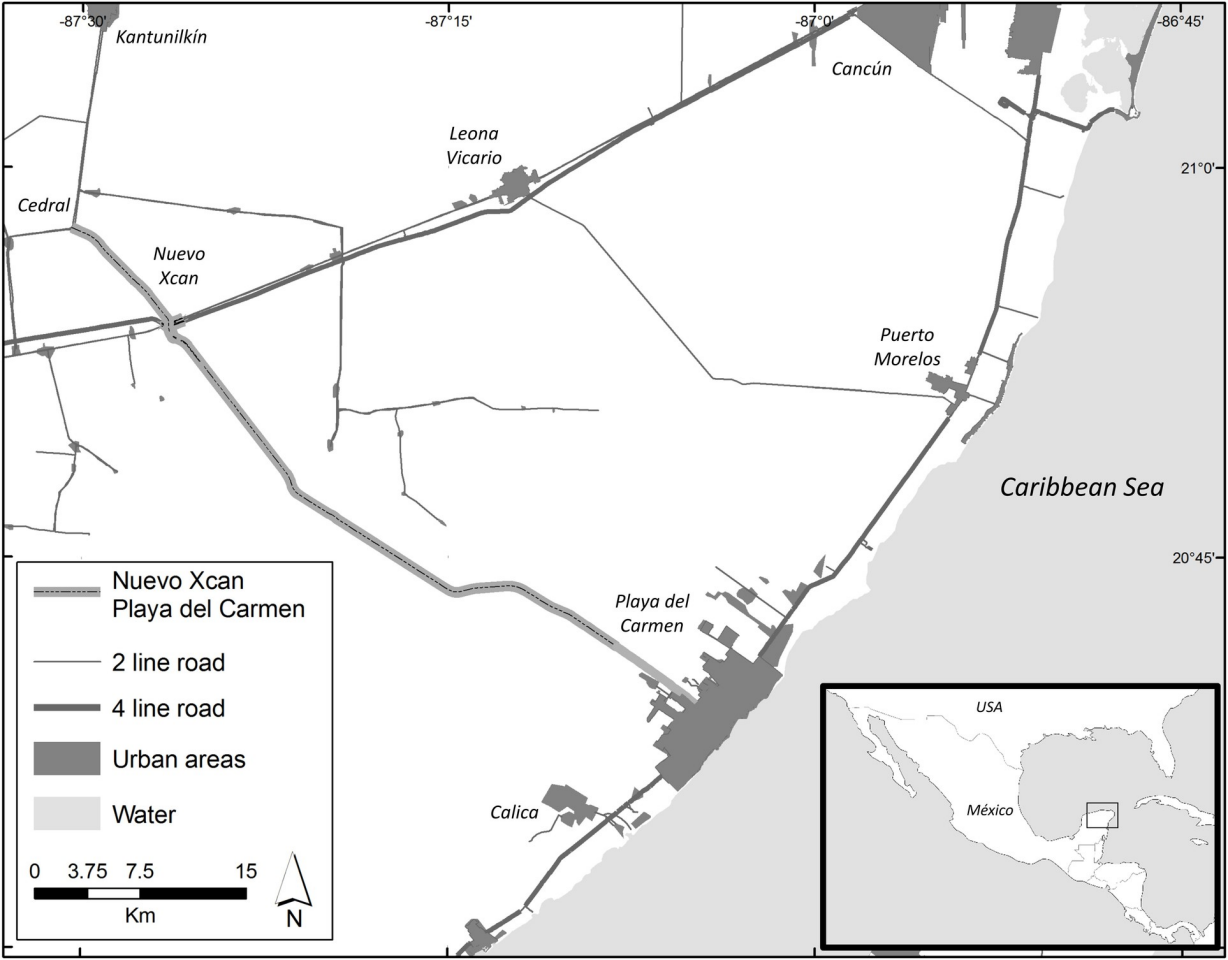
The Nuevo Xcan- Playa del Carmen highway in Quintana Roo state, Mexico. The NX-PC highway starts in Luis Donaldo Colosio Avenue in the city of Playa del Carmen and bears primarily North East 54km until it terminates at the Cedral junction with Holbox highway. It is a type A2 highway with two lanes with one circulation lane per direction with wide shoulders in which vehicles may circulate with a cruising speed of 110km/h.

The NX-PC highway (Fig 1) is located in the northeastern portion of the Yucatan Peninsula in Mexico. The highway runs through the municipalities of Solidaridad and Lazaro Cardenas in the state of Quintana Roo. The area is mostly flat with an altitude of 5–10 m a.s.l. Weather is warm and sub-humid with annual mean temperatures as low as 26°C and as high as 33°C. Annual mean rainfall is 1,300mm and normally concentrates around the months of June to October [43]. Natural vegetation of the area is moist forest (selva mediana subperenifolia [44]; however due to the recurrence of hurricanes, agricultural burns, and urbanization of the area is now covered mostly with secondary forests in different succession stages, temporary agricultural areas and peri-urban areas [45]. The landscape is dominated by mature second growth forests (with approximately 25 years of regeneration after hurricane Gilbert in 1988), these characterized by trees with a canopy height of 8-10m and a closed understory and younger secondary growth forest (around 10 years of regeneration), dominated by shrub and herbaceous strata as result of recent induced fires. Some areas are open for agriculture with slash and burn techniques, commonly abandoned after several years of use. Cattle grazing is constrained to a few cropland areas and introduced grasslands for this purpose. The study area is located from the outskirts of Playa del Carmen city, where the tourism industry has been the main driver of development to the North East. As the top tourist area of southern Mexico, Playa del Carmen has experienced some of the most rapid urbanization in the world, with mean annual population growths reaching up to 20.5% a year [46–47].

The highway project was originally named “Ramales Cedral-Tintal y Tintal-Playa del Carmen con una longitud de 54km en el Estado de Quintana Roo” by Mexican authorities. Ingenieros Civiles y Asociados Infrastructure Division (ICAi) through its subsidiary Consorcio del Mayab were in charge of building and operating the road; which began operations on September 2014. The highway starts in the Luis Donaldo Colosio avenue within Playa del Carmen, bearing North East, ending in the Cedral junction with Holbox highway (Fig 1). Classified as an A2 highway by the Mexican authorities (12m wide road with one circulation lane per direction, wide shoulders designed for cruising speeds of 110km/h [48]. The highway had an average daily transit of 1,533 vehicles during 2016 [49].

**Fig 2:**
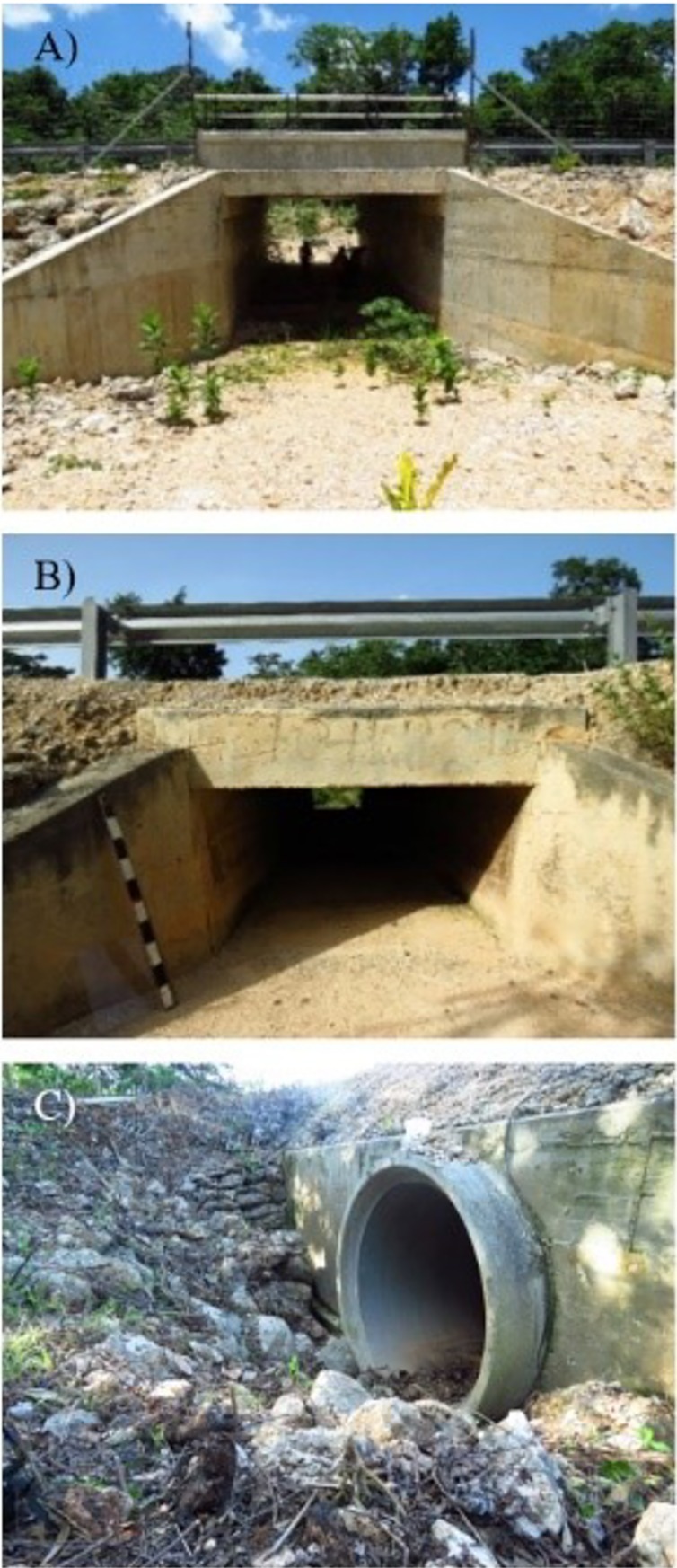
Crossing structure types build along the Nuevo Xcan- Playa del Carmen highway in Quintana Roo, Mexico. A.- Wildlife underpasses (3 m span x 4.5m rise, equipped with induction fences, natural soil, and without barb wires delimiting the right of way). B.- Box culverts (2 m span x 1 m rise). C.- Concrete pipes (circular ducts with a diameter of 1.8 m). The box culverts and the concrete pipes were not built with specific mitigation measures for wildlife crossing.

The highway includes 28 Wildlife Underpasses (WU) along its length as a mitigation measure to maintain the connectivity for jaguars and facilitate the safe crossing of wildlife. The WU are concrete structures 3 m span and 4.5m rise with natural soil. The underpasses are repeated every 200 m to 2.5 km (mean 1200 m; Fig 2). Each WU has induction fences, 2 m high, on both sides of the road that run about 100 m on each direction to direct wildlife. Also, the typical barbed wire fence that delimitates the right of way along most of the highway were removed around the underpass areas but for upper wire to provide easier access to wildlife. To avoid obstruction of the WU, both entrances and interior of the structures are specifically cleared of vegetation (herbaceous and shrubs), falling rocks and trunks and trash at least once every two months by road maintenance crews.

Along the 54 km of the NX-PC road, culverts consist of 32 concrete box culverts (BC) and 56 concrete pipes (CP). The BC are 2m span x 1m rise (Fig 2). The CP are circular ducts with a diameter of 1.8 m (Fig 2). Neither of the culverts were built as specific mitigation measures for wildlife. They are not equipped with induction fence and the barb wire on the right of way is left intact. Road maintenance crews regularly clear the entrance of these culverts of vegetation as a part of the regular cleaning of the right of way, while their interiors are only cleaned if there are obstructions impacting proper drainage.

### Monitoring of wildlife underpasses and culverts

We established 28 monitoring stations that continuously operated from July 2016 to July 2017, ten stations on WU, nine each on BC and CP. Each station consisted of a single camera trap (Pantheracam V. 6.) programmed to operate 24 hours per day and to obtain 3 images per trigger event. Due to previous experiences of equipment theft along the highway and the limited number of available cameras, our first criteria for selecting monitoring station locations was selecting those areas with reduced human activity (external to highway maintenance crews). Once we determined these areas, we distributed the camera traps along the structures where forest vegetation was dominant (including moist forest and secondary growth forests), where jaguar presence was more likely to occur [31].

In WU, cameras where set in the middle section inside the structure, perpendicular to the entrance aimed to photograph all passing animals. Camera were installed approximately 50 cm from the ground by drilling and placing plastic plugs and after fixing the cameras to the wall using perforated metallic tape with screws. In BC and CP due to design and size as well as the lack of proper fixing places, we decided to place the cameras in the entrance edge in a 45° angle facing interior of the structures. A single camera was placed at each station. We checked operation of all the camera stations monthly, in which we also changed the batteries and downloaded the photographs.

Permission to work along the Nuevo Xcan-Playa del Carmen highway was granted by Ingenieros Civiles Asociados (ICA) to whom the Mexican government and the transportation authorities (SCT) concession the operation of the highway. No specific permit was required or issued by the Mexican environmental authorities (SEMARNAT) because the study area took place outside any natural protected areas. The project did not require any manipulation of organisms of any kind due to non-invasive photo trapping techniques, even when it involves some species under protection by Mexican law within the NOM-059-SEMARNAT-2010.

### Data analysis

Photographs were stored and processed using Camera Base 1.7 [50]. We excluded all non-mammalian taxa. We identified species using Reid (2009) [51]. We considered independent events to be consecutive photographs of the same species separated by at least one hour [52]. We used independent photographic events as an approximation of the use of each species. Sampling effort per station was obtained by counting the days from when the camera was activated to the date of the last photograph taken subtracting days when the camera was not functioning. We considered a camera day to be a period of 24 hours during which the camera was operating and total sampling effort as the added number of camera days that each camera station operated on each structure [53].

In the case of jaguars, we identified them individually based on their skin pattern through visual examination [54–55]. Identification of individuals using the structures was based on a single side [56]. When possible, we determined the sex of the jaguars from the photographs.

We calculated alpha and beta diversity with the independent events obtained by structure type: WU, BC and CP. For some analyses, we merged both culvert types (BC and CP) into a single category called culverts (C). In Latin America, many highway builders have claimed culverts function as proper wildlife underpasses even with no retrofitting. True diversity indexes (^q^D sensu [57]) for all different orders q = ^0^D (species richness, Sobs), ^1^D (Shannon´s exponential index, e^H^) and ^2^D (Inverse Simpson´s index, 1/D), were calculated separately for each station and grouped by structure type [58] (S1 Data). We compared mammal diversity of the different crossing structures using iNext software [59]. We estimated diversities for standardized samples for equally-large (common sample size) or equally-complete (common sample coverage) based on the seamless rarefaction and extrapolation sampling curves of True diversity indexes for q = 0, 1 and 2 [58, 60–61]. We obtained asymptotic diversity profiles based on statistical estimation of the true Hill number of any order q > = 0 [62]. To determine if there were differences among the size of the mammal species using the different crossing structures we calculated the aggregated biomass by adding the species average weight (kg) reported from the literature [51] of all mammal species photographed on each station [63] (S1 Data). Then we grouped all stations by structure type and compared the total weight per structure using a Kruskal-Wallis analysis. A Dunn´s post hoc test was run [64] if the medians were found to be different.

Beta diversity (changes in species composition) was assessed between crossing structures using Bray-Curtis index (I_BC_ [65]) with the following formula:

$$I_{BC}=\frac{2∙N_{ab}}{N_{a}+N_{b}}$$

Where Na = Total number of independent events per structure A and Nb = Total number of independent events for structure B, with Nab = Sum of the minimum abundances for each of the shared species between structure type. I_BC_ with a value of 1 means species composition between both structures were identical, and the closer to cero the more different they were.

Bats and small, non-identifiable rodents were excluded from the diversity and structures analyses, as both comprise several species that could bias the results. We also excluded from the analyses domestic dogs *Canis familiaris* and cats *Felis catus)* to properly assess the use of the structure by the native mammal community. Opossums *Didelphis virginiana* and *D*. *marsupialis*, were grouped in the category *Didelphis* spp.

## Results

**Fig 3:**
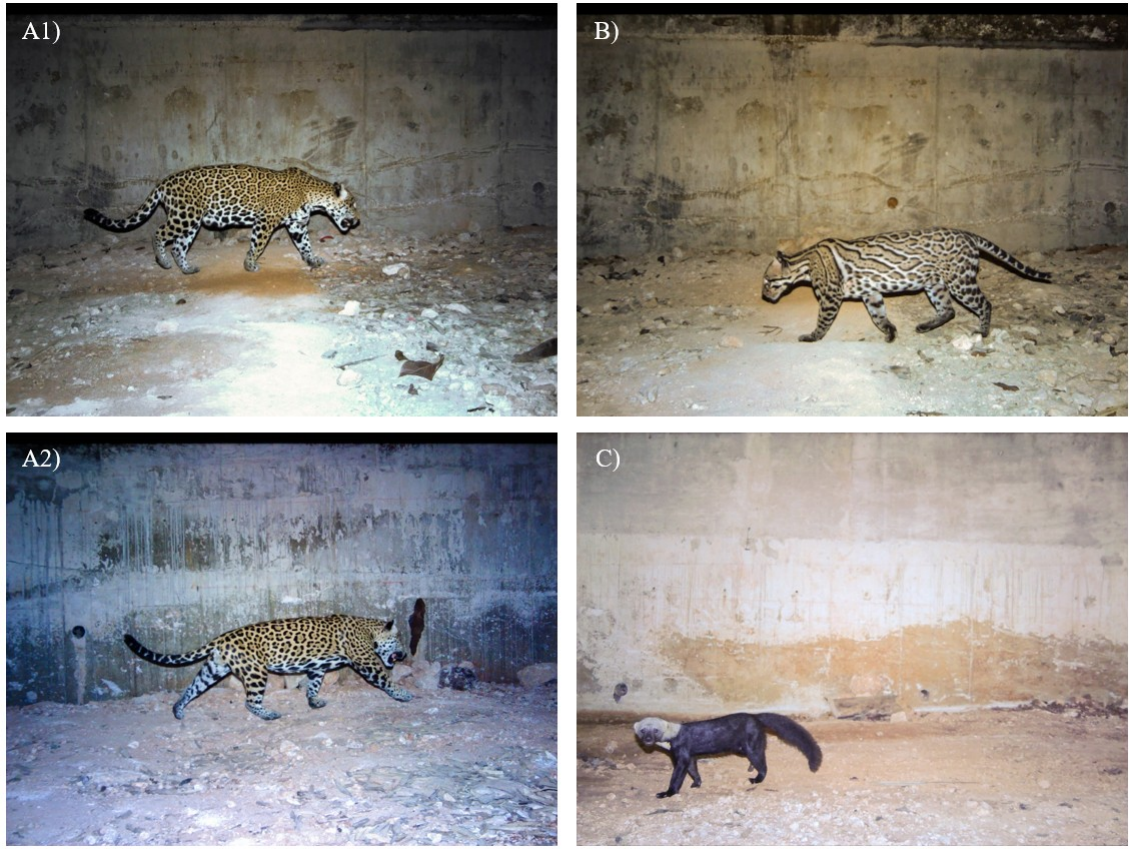
Photographs from priority species of mammals using wildlife underpasses. Priority species considered as Endangered (P) according to Mexican law (NOM-059-SEMARNAT-2010). A) Jaguars (*Panthera onca*) using two different stations, 1) female and 2) male. B) Ocelot (*Leopardus pardalis*) and C) Tayra (*Eira barbara*).

From July 2016 to July 2017 we had a total sampling effort of 10,166 camera days for all the structures (3,630 in WU, 3,268 in BC and 3,268 in CP) and obtained 2,559 independent events (653 in WU, 1307 in BC and 611 in CP) from at least 15 native mammal species (Table I). The observed species richness was highest in the WU (12 species), followed by the CP (nine species), and the BC (six species). Six species are under protection by Mexican law (NOM-059-SEMARNAT-2010), five as endangered (P) and one as the threatened (A). Five of the six protected species were observed on the WU (Fig 3), three on the CP and two on the BC (Table 1). Also, we recorded two domestic species using the structures (domestic dogs and cats). Human presence was recorded in 24 out of the 28 surveyed structures and consisted mainly of highway maintenance crews clearing vegetation (63 records), and local inhabitants (13 records). We performed the survey in only 13.7% of all the available structures in the highway, (35% of the WU, 20.4% of culverts meaning 28% of BC and 16% of CP) so our results are underestimations of the full extent of the usage of the wildlife crossings through this highway.

**Table 1:** Mammal use of crossing structures along the Nuevo Xcan-Playa del Carmen Highway, Quintana Roo, Mexico (July 2016- July 2017). Species are taxonomically ordered by order, family and species (Ramírez-Pulido et al. 2014 [66]) with their common names and protection status. Independent events are shown by species per crossing structure type: wildlife underpasses (WU), box culverts (BC) and concrete pipes (CP), culverts (C) and total records. The numbers between parenthesis are the number of stations where species were recorded.

	Species	Common Name	NOM^a^	WU^b^	BC^b^	CP^b^	C^b^	Total Number of Records^b^
Didelphimorphia								
Didelphidae								
	*Didelphis spp*.	Oposum		9 (7)	19 (5)	5 (4)	24 (9)	33 (16)
Chiroptera								
	*Chiroptera*	Bats		93 (9)	1185 (9)	480 (9)	1665 (18)	1749 (27)
**Primates**								
Atelidae								
	*Ateles geoffroyi*	Spider monkey	P	1 (1)				1 (1)
**Rodentia**								
Sciuridae								
	*Sciurus yucatanensis*	Yucatan squirrel		1 (1)		3 (2)	3 (2)	4 (3)
Agoutidae								
	*Dasyprocta punctata*	Agouti		184 (3)				184 (3)
Cuniculidae								
	*Cuniculus paca*	Paca		124 (6)	7 (2)	40 (4)	47 (6)	171 (12)
Cricetidae								
	*Cricetidae*	Rats		5 (5)	4 (2)	23 (3)	27 (5)	32 (10)
**Carnivora**								
Felidae								
	*Felis catus*^*c*^	Domestic cat		1 (1)	1 (1)		1 (1)	2 (2)
	*Leopardus pardalis*	Ocelot	P	12 (3)		2 (2)	2 (2)	14 (5)
	*Leopardus wiedii*	Margay	P		1 (1)	2 (1)	3 (2)	3 (2)
	*Panthera onca*	Jaguar	P	24 (7)				24 (7)
Canidae								
	*Canis familiaris*^*c*^	Domestic dog		1 (1)	1 (1)		1 (1)	2 (2)
	*Urocyon cinereoargenteus*	Grey Fox		181 (9)	79 (7)	49 (8)	128 (15)	307 (24)
Mustelidae								
	*Eira barbara*	Tayra	P	8 (4)	7 (1)		7 (1)	14 (4)
	*Galictis vittata*	Greater grison	A	5 (2)		2 (2)	2 (2)	7 (4)
	*Mustela frenata*	Long tailed weasel				3 (2)	3 (2)	3 (2)
Procyonidae								
	*Nasua narica*	Coati		3 (2)		2 (2)	2 (2)	5 (4)
	*Procyon lotor*	Racoon			3 (2)		3(2)	3 (2)
**Arctiodactyla**								
Cervidae								
	*Odocoileus virginianus*	White-tailed deer		1 (1)				1 (1)
		Total records		653	1307	611	1918	2559

^a^ Status according to the Mexican Endangered and Protected Species Legislation. P. Endangered (en peligro de extinción); A. Threatened (amenazada)

^b^ Number in parenthesis indicates the number of structures where the species was recorded

^c^ Domestic species

The most recorded mammals were the bats (various unidentified species, 1,749 records), followed by the gray fox (*Urocyon cinereoargenteus*, 307 records), the agouti (*Dasyprocta punctata*, 184 records), the paca (*Cuniculus paca*, 171 records) and the opossum (*Didelphis spp*., 33 records; Table 1). Besides bats and small rodents, only three species used all three types of structures (grey fox, paca and opossum; Table 1), six used two structures (Yucatan squirrel, ocelot, margay, tayra, greater grison and coati) and six were recorded in a single structure type, four of them using WU exclusively (spider monkey, agoutis, jaguars and white-tailed deer), and one using the using BC (raccoon) and one the CP (long tailed weasel). In the case of the jaguars, we recorded 24 independent jaguar events during the study (all of them in WU). They were recorded on seven of the ten monitored underpasses. We identified at least six individual jaguars were using the WU (two females and four males).

**Fig 4:**
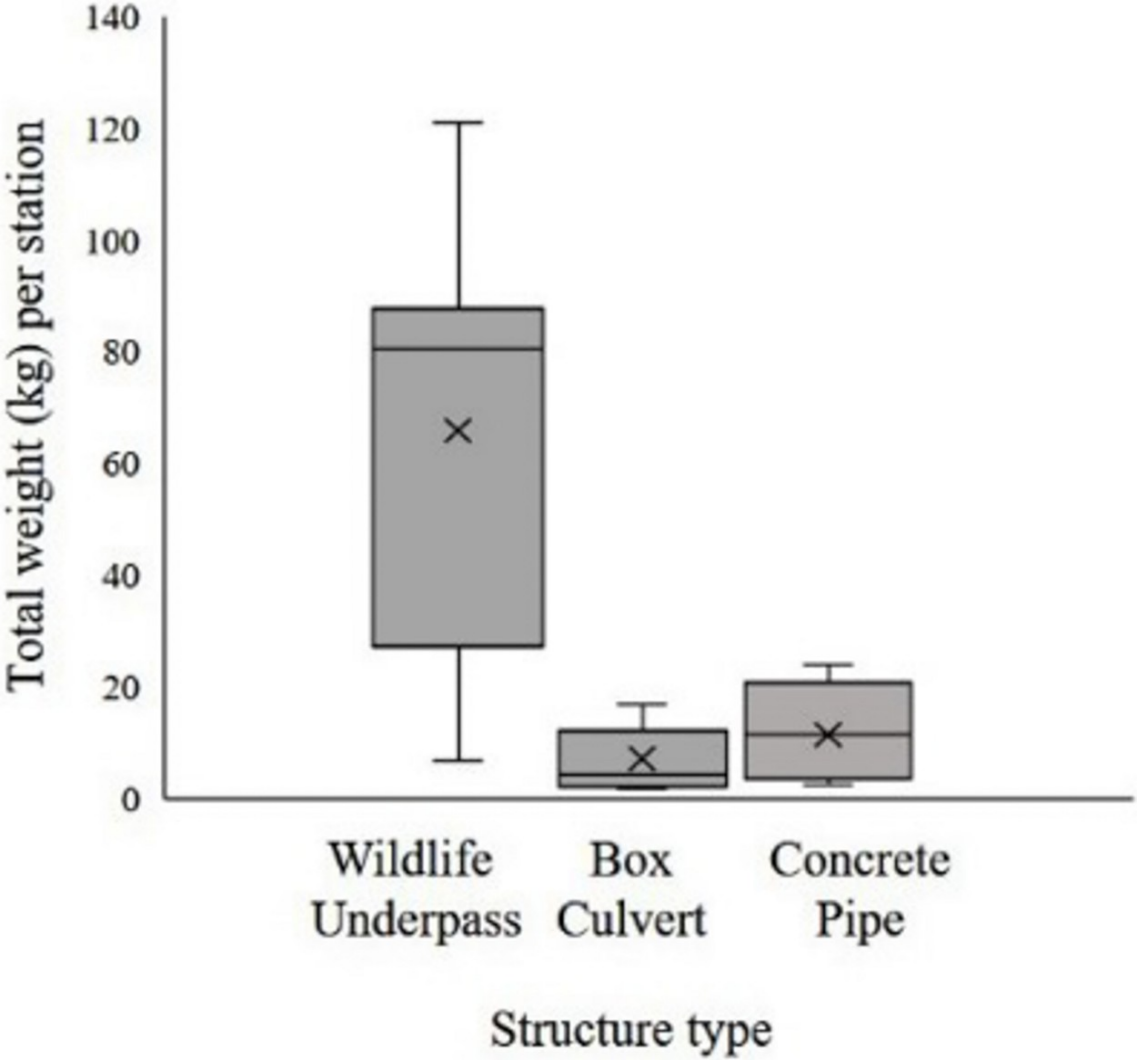
Weight of the mammal species using each station by crossing structure type. Aggregated biomass in kg per species (in Reid 2009) using each station. Mean appears as line dividing the box, whiskers represent 95% confidence intervals.

When we included the sum of the average weights of each species using the different crossing structures in the analysis (Fig 4, S1 Data), the WU had a mean weight of 83 kg against those in the culverts with BC 6.9kg and CP 11.33kg. The Kruskal-Wallis test between all structures showed very strong evidence of a difference (H = 13.43, p = 0.001, df = 27). Dunn´s post-hoc test showed WU showed no substantial difference when compared to culverts (C) with a value of 0.4 but it became significantly different when compared to each culvert separately, with values for BC = 0.0004 and for CP = 0.007.

**Fig 5:**
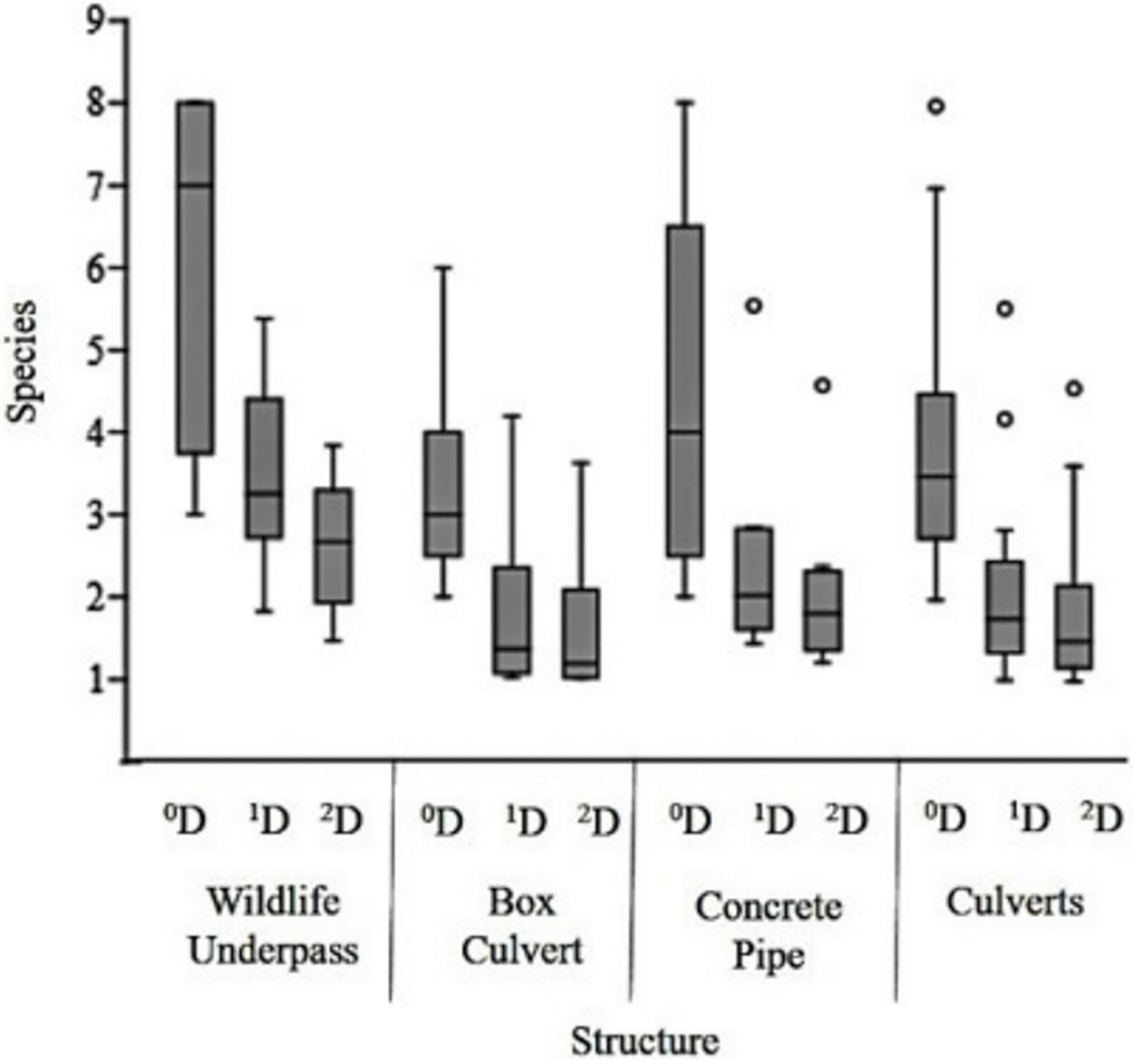
Individual station diversity values per structure type. All crossing structures: wildlife underpasses, box culverts, concrete pipes and both culverts grouped, showing True diversity indexes (^q^D) for all levels ^0^D (species richness), ^1^D (Shannon´s exponential) and ^2^D (Inverse Simpson). Mean appears as a line dividing the box, whiskers represent 95% confidence interval. Outlier values are shown as circles.

**Fig 6:**
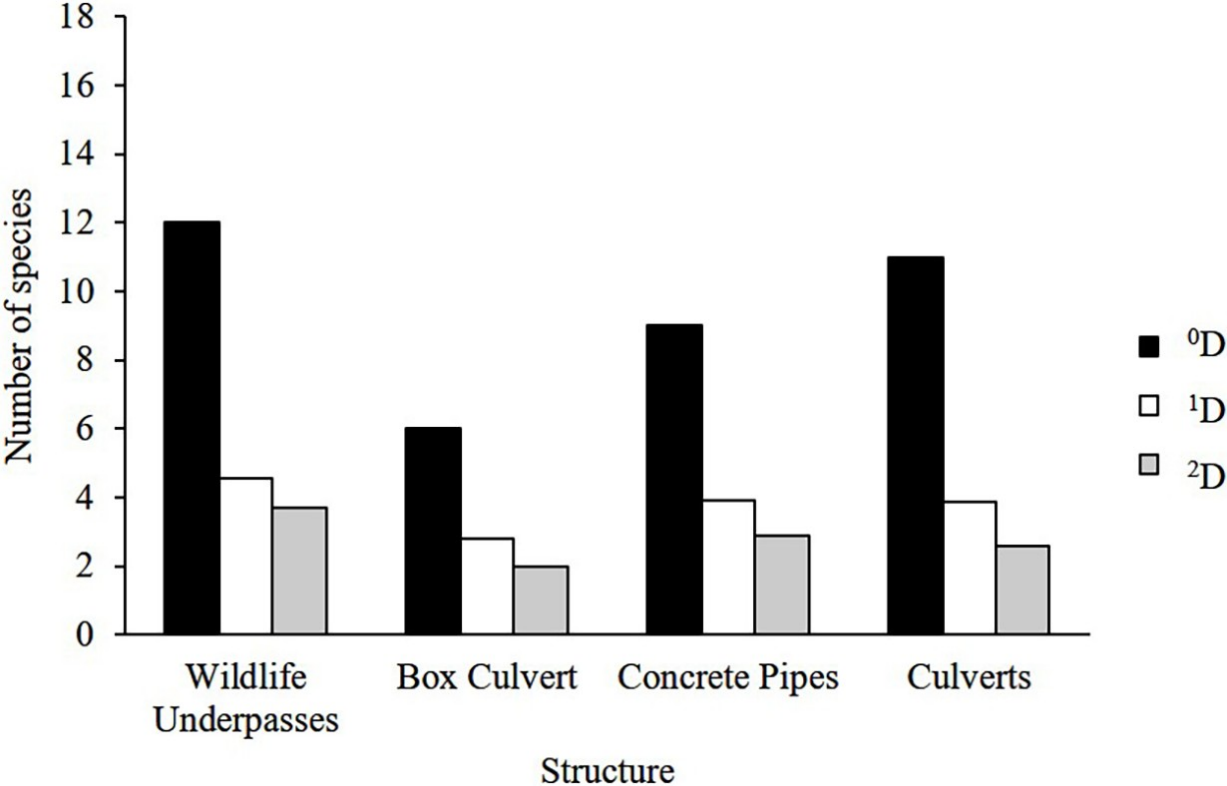
Terrestrial mammal diversity profile for the different crossing structures along the Nuevo Xcan- Playa del Carmen highway. All crossing structures: wildlife underpasses, box culverts and concrete pipes and culverts together. True diversity indexes (^q^D) for all levels ^0^D (species richness), ^1^D (Shannon´s exponential) and ^2^D (Inverse Simpson).

**Fig 7:**
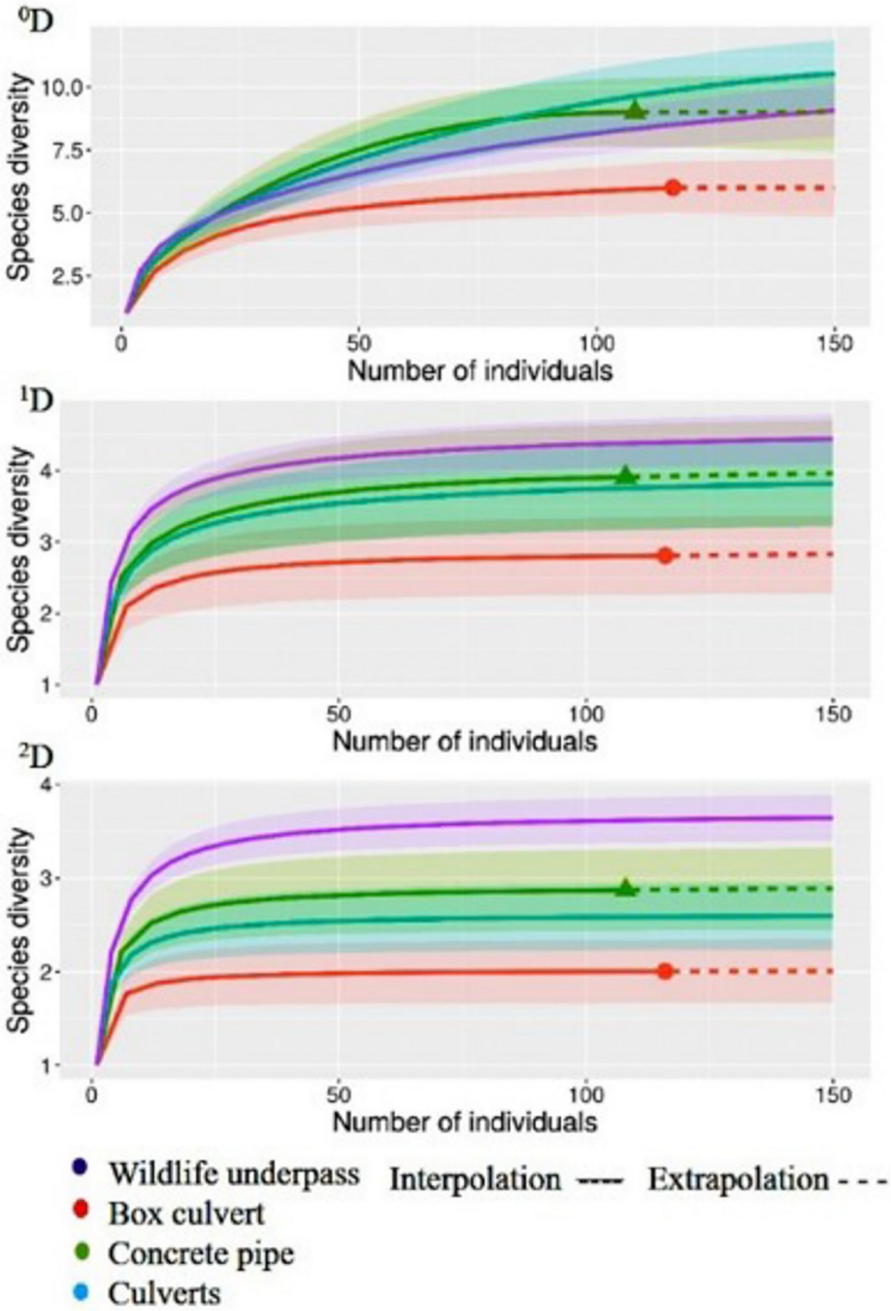
Species accumulation curves for the three types of wildlife crossing structures monitored structures along the Nuevo Xcan- Playa del Carmen highway in Quintana Roo, Mexico. Accumulation curves were obtained by using true diversity indexes (^q^D) at three levels, ^0^D (species richness), ^1^D (Shannon´s exponential) and ^2^D (Inverse Simpson). Continuous lines are the recorded richness curves, and discontinuous lines are confidence intervals.

After excluding the introduced species, bats and small rodents from the analysis, we recorded 553 individuals in the WU compared with the 116 in the BC and 108 of the CP. The mean number of species (^1^D) per camera station (Fig 5) was 3.42 for the WU (1.83 min -5.38 max) 1.8 for the BC (1.03 min- 4.2 max), 2.43 for the CP (1.43 min- 5.54 max) and 2.1 for Culverts (1.03 min– 5.54 max). When assessed by structure (Fig 6), WU had higher mean values (^1^D = 4.57, ^2^D = 3.7) than culverts compared to when they are assessed together (^1^D = 3.8, 1/D = 2.6) and separated with BC (^1^D = 2.81, ^2^D = 2.01) and CP (^1^D = 3.91, ^2^D = 2.87). This trend is also observed in the rarefaction curves, where we observed true diversity values (^q^D) at all levels ^0^D, ^1^D and ^2^D to be higher for WU than culverts, followed by CP and BC (Fig 7). The results for true diversity per structure type showed that the number of species captured was 99% of that predicted by the sample coverage estimate (S1 Fig). All data used for true diversity analysis per station and per structure type are available in S1 Data. Based on sampling efficiency, the data appears to be a reliable sample of the mammal diversity for each structure.

For beta diversity we found that the Bray-Curtis index showed that the species composition between the three structures was different. When compared with each other, BC and CP had the most similar species composition (0.55), followed by the comparison between WU and Culverts (0.51). We observed that the species composition between the WU with both the BC (0.3) and CP (0.31) individually had the lowest species similarity values on the Bray-Curtis index.

## Discussion

### Mammal use of crossing structures in the NX-PC

We found differential use of crossing structures by mammals, in agreement with other studies worldwide (reviews in: [13, 67–68]). Taxonomic groupings and body size may act as partial surrogates for an animal’s ability and willingness to use a crossing structure, as similar species may share certain characteristics with regards to size, means of locomotion, and environmental constraints. However, taxonomic groups do not reliably account for ecological adaptations (based on their movement, behavior and physiological needs) that influence crossing structure use [36].

Ecological traits [36] can cause some mammals to become recognized as either structure specialists, using a single structure type, or structure generalists, by using all structures. Out of six specialists, four used only WU including the spider monkey, the jaguar, white-tailed deer and agoutis. The other two used only culverts with the raccoon using only BC and the weasel CP. A clear example is the regular presence of pacas in all structure types. Pacas are nocturnal and live in burrows [51] which makes them prone to use “artificial caverns” such as the underpasses and drainages more often than other species. Agoutis are in the same taxa as Pacas but show different trends. Agoutis only used wildlife underpasses and avoided smaller structures despite being about 5kg smaller than pacas. This suggests that rather than size, what determines their use of crossing structures seems to be their diurnal habits [51], which translates into them preferring structures with more light.

Sparks and Gates (2017) [69] state that results from this kind of surveys are actually a measure of activity; however, whether it is 10 individuals crossing once or 1 individual crossing 10 times, the end result is nonetheless a reduced likelihood of becoming road kill. For abundant species (coatis, raccoons, opossums, etc.), using structures less frequently than expected compared to other studies [32], crossing structures appear to be less than adequate which could be reflected in an increased number of wildlife-vehicle collision events. Habitat generalists [51] are more susceptible to road mortality than to the other effects of roads [69]. Some species that are common across the region [32] and also commonly observed using structures are the grey fox and the pacas, both in all structure types and the agoutis in the WU. Grey foxes are the most common users of all structures besides bats.

Our findings using species rarefaction curves suggest that our monitoring period was sufficient to find use patterns in the different structures. Considering monitoring occurred three years after the highway began operation (September 2014), likely long enough for wildlife to learn and adapt to the disturbance and the crossing structures [70]. Some mid-sized, local species were not recorded at all [32, 71], such as armadillos (*Dasypus novemcinctus*), jaguarundi (*Herpailurus yagouaroundi*), skunks (*Conepatus semistriatus* and *Spilogale angustifrons*), peccaries (*Tayassu pecari*) and brocket deer (*Mazama pandora*, *M*. *temama*) [51]. Several of these species have been reported using underpasses elsewhere [72] suggesting it may just be a matter of time before they are observed using the structures here, considering we didn´t cover all available structures.

### Wildlife underpasses: Proper crossing structures

The WU were built considering several priority species [30], the ocelot, margay, jaguar and the tayra. These species were selected for their conservation status, as most are considered to be endangered (P) by Mexican law (NOM-059-SEMARNAT-2010). Monitoring to investigate if the target species are using the structures is a critical first step although it does not provide a complete insight into the effectiveness of a structure [73].

In the case of jaguars, the WU appear adequate. We documented at least six individuals, of both sexes, using multiple structures on multiple occasions. Avila-Nájera (2015) [31] estimated 5.5 jaguars/100km^2^ in the region, indicating that the observed number of individuals crossing in the WU could be an important part of the jaguar population inhabiting the nearby area. Success of a wildlife crossing structure not only depends on the design, there are other variables that should be evaluated to find the reasons why some structures fail. For instance, we did not record any crossing in three of the WU. There is evidence that carnivores do not cross highways randomly, but rather focus their crossing activity to locations that vary in passage characteristics, road-related attributes, surrounding habitat characteristics, and human disturbance levels [67, 74–75].

Pumas were also considered target species not because of their conservation status (least concern, Pr) but because they are the second largest carnivore in the area [32]. They only used WU, the same as jaguars. WU were designed in a similar fashion to those used by the species in other places [i.e., 40]. It is noteworthy that no puma records where attained during this particular survey despite being present in the area [76]. However, during monitoring conducted by the consultancy (SEGA S.A. de C.V.), they recorded two puma crossings while monitoring 27 WU (all except for one in a wetland). Another potential reason we did not record puma use, is that threat of theft and actual loss of equipment prevented some WU from being monitored. Finally, it’s possible that the low crossing rates by pumas suggests they may prefer crossing over the highway as they are more prone to use open spaces than jaguars [77–78].

Almost all target species used WU but not exclusively, except for jaguars. Ocelots and tayras were recorded in WU and some culverts. In the case of the margay, another target species for which the WU should be working, we have no records but sporadic crossings in the culverts. We have witnessed that WU provide occasional crossings for other species such as deer and spider monkeys. Monkeys are first recorded crossing this highway underneath it, rather than over it in one of the 22 canopy rope bridges built for arboreal species.

Wildlife fencing in combination with crossing structures is commonly regarded as the most effective and robust strategy to reduce large mammal–vehicle collisions while also maintaining wildlife connectivity across roads [79]. Fence length varied along the NXPC from about 100m on each side to about 3km. As stated by Huijser et al. [79], the presence of wildlife fencing and longer fence lengths can improve but do not necessarily guarantee higher wildlife use of underpasses. Further studies are required to determine the precise effect of fencing in this particular setting and, more broadly, in the Neotropics. We are still lacking roadkill surveys of this highway and its neighboring non-mitigated highways to assess wildlife vehicle collisions, which also could be used to compared fenced and non-fenced areas to this matter.

### Culverts: Accessory crossing structures

The number of species (^0^D) in the WU is greater than any other individual structure (BC or CP). However, when grouping CP and BC as drainage, the diversity behavior begins to look like the one in the WU in terms of observed species. Nevertheless, values for shared species are low between WU and other structures which indicates that different species go through the WU than the ones using drainages. WU clearly allows bigger (heavier) species to cross compared to drainages (e. g. carnivores [37]; herbivores [80]). When comparing culverts, both BC and CP have similar species composition despite that BC appears with lower values in this case. Values of ^2^D show that the WU are used less amongst species in comparison to what happens in culverts, BC yields the highest species dominance.

Despite that Mexico has culverts in many highways that could potentially function as wildlife underpasses, this by no means should substitute the value of properly built wildlife crossing structures such as the studied WU. When viewed as individual stations, only few culverts (perhaps due to location) achieved diversity values as high as WU although in average they cover less species. This already means that culverts in the right place can be used by many species [39, 75, 81]. These drainage culverts are a standard procedure during highway construction and should be retrofitted to enhance their value as wildlife crossing structures [82], this could be achieved by adding drift fences in the way the WU have, with adaptations for smaller species [83], in order to reduce wildlife vehicle collisions and improve wildlife use [78]. Hence, the importance of counting with several structure types to allow for the flow of a wide diversity of wildlife and thus maintain connectivity [12,67].

### Implications for jaguar conservation

The NX-PC project shows that wildlife underpasses can be built across highways to maintain functional connectivity among jaguar populations.

Large carnivores such as jaguars are important umbrella species whose conservation can contribute to the maintenance of co-occurring mammal species [84]. There is substantial evidence demonstrating that a reduction in apex predators can alter composition, structure and functionality of entire ecosystems [85–86]. Road development-induced ecological impacts also undoubtedly extend beyond population-level effects on jaguar and prey [22]. Within the Mayan forests, proximity to roads reduces the probability of occurrence for jaguars, with males showing higher tolerance to roads than females [23]. This also affected their movements, with females being much more restricted than males at even intermediate levels of human population densities [24]. Figueroa (2013) [87] found than even though jaguars tolerate dirt roads, there is a strong avoidance when it comes to paved roads with increased vehicular traffic. Our results of both male and female jaguars crossing underneath the highway through WU makes the value of such mitigation measures evident for maintaining connectivity between jaguar populations. It also makes us question if what we knew previously about jaguars and roads applies only within natural protected areas where jaguars can choose whether to come close to roads or not and if outside of protected areas, the behavioral plasticity of such animals make some of them more daring than others.

While WU have proven useful for maintaining connectivity between the jaguar population, they do not seem to be so significant in maintaining connectivity between the populations of their main prey [76, 88–89], except for big rodents (pacas and agoutis). An important consideration with large herbivores in their role as prey is that they need to avoid predators when using crossing structures. Structures that are more open than confined work better for these species [79]. Ungulates (white tailed deer, brocket deer and peccary) that represent big prey for jaguars in the area [76] may require a wider span than the one available in the constructed underpasses (only 3m). So far only a couple of young white tail deer males have used the WU, and although peccaries have been reported using drainage as crossing structures they have not used the ones we monitored.

Highways sometimes work as corridors for invasive species [68] including with domestic cats and dogs. Domestic dogs are prey for jaguars [90] and although they were not frequent in monitored underpasses (except? next to human settlements), functional underpasses that allow jaguar movement may enhance dog as an available resource.

Road agencies around the world are responding to the changes that society is demanding by including greater consideration of ecological issues when planning, building and managing road networks. It is imperative that road agencies successfully adapt to these changes to ensure the future road network is as environmentally friendly as possible [91]. In the study area, the NX-PC highway is one of five that cut the vegetation corridor between Yum Balam (north) and Sian Ka´an (south) JCUs, and the only one with proper mitigation measures for maintaining connectivity (at least for mammals).

For a corridor of any significant scale to have a chance at success and sustainability, conservation practitioners must negotiate a maze of land tenure, land use, jurisdiction issues, and legal issues before deciding upon strategies and approaches. Each corridor has its own unique set of circumstances, threats, and opportunities that need to be addressed for success to occur. Long-term financial and political commitments are a key component of the process [14]. It is our hope that culverts along these other highways could be retrofitted and turned into wildlife crossing structures and that some WU are built to maintain movements and long term connectivity for jaguars and the rest of wildlife in the area. Yet, further conservation actions, such as the construction of WU, are needed along the corridor and throughout the region. If there is not sufficient habitat left, wildlife corridors and crossing structures will be useless [92] as there will be no wildlife remaining.

## Supporting information

S1 Data Datasets for mammal true diversity analysis per station and structure type, and weights of mammal species using each station by structure type. (XLSX)

S1 Fig Terrestrial mammal sample completeness curves for the monitoring of the different crossing structures: wildlife underpasses (WU), box culverts (BC), concrete pipes (CP) and both culverts together along the Nuevo Xcan-Playa del Carmen highway. (TIF)
