# Supplementary figures and images for: Conservation implications for jaguars and other neotropical mammals using highway underpasses
Source: PLoS One. 2018 Nov 6;13(11):e0206614. doi: 10.1371/journal.pone.0206614 (PMC6219781; doi:10.1371/journal.pone.0206614)

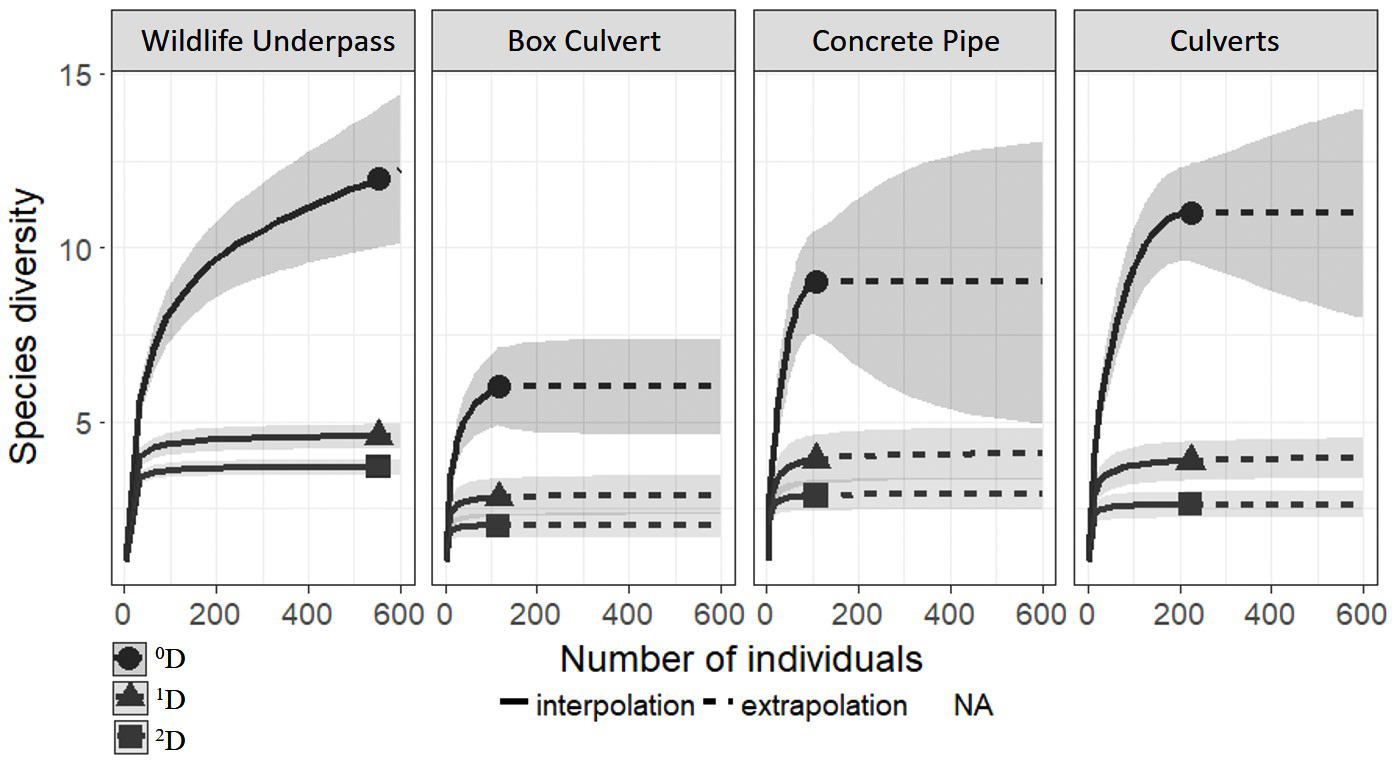

Supplement: S1 Fig — (TIF) [file pone.0206614.s002.tif]
